# Geographic and socioeconomic variation in treatment of elderly prostate cancer patients in Norway – a national register-based study

**DOI:** 10.1007/s43999-024-00044-y

**Published:** 2024-05-15

**Authors:** Elin Marthinussen Gustavsen, Erik Skaaheim Haug, Ellinor Haukland, Ragnhild Heimdal, Eva Stensland, Tor Åge Myklebust, Beate Hauglann

**Affiliations:** 1 Department of Community Medicine, The Arctic University of Norway (UiT), Tromsø, Norway; 2 Centre for Clinical Documentation and Evaluation (SKDE), Northern Norway Regional Health Authority, Tromsø, Norway; 3 Department of Urology, Vestfold Hospital Trust, Tønsberg, Norway; 4 Department of Oncology and Palliative Medicine, Nordland Hospital, Bodø, Norway; 5 SHARE – Center for Resilience in Healthcare, Faculty of Health Sciences, Department of Quality and Health Technology, University of Stavanger, Stavanger, Norway; 6 Geriatric Department, Akershus University Hospital, Lørenskog, Norway; 7 Cancer Registry of Norway, Norwegian Institute of Public Health, Oslo, Norway; 8 Department of Research and Innovation, Møre and Romsdal Hospital Trust, Ålesund, Norway

**Keywords:** Geographic variation, Socioeconomic variation, Prostate cancer, Elderly patients, Health care utilisation

## Abstract

**Purpose:**

The aim of this study was to examine geographic and socioeconomic variation in curative treatment and choice of treatment modality among elderly prostate cancer (PCa) patients.

**Methods:**

This register-based cohort study included all Norwegian men ≥ 70 years when diagnosed with non-metastatic, high-risk PCa in 2011–2020 (*n* = 10 807). Individual data were obtained from the Cancer Registry of Norway, the Norwegian Prostate Cancer Registry, and Statistics Norway. Multilevel logistic regression analysis was used to model variation across hospital referral areas (HRAs), incorporating clinical, demographic and socioeconomic factors.

**Results:**

Overall, 5186 (48%) patients received curative treatment (radical prostatectomy (RP) (*n* = 1560) or radiotherapy (*n* = 3626)). Geographic variation was found for both curative treatment (odds ratio 0.39–2.19) and choice of treatment modality (odds ratio 0.10–2.45). Odds of curative treatment increased with increasing income and education, and decreased for patients living alone, and with increasing age and frailty. Patients with higher income had higher odds of receiving RP compared to radiotherapy.

**Conclusions:**

This study showed geographic and socioeconomic variation in treatment of elderly patients with non-metastatic, high-risk PCa, both in relation to overall curative treatment and choice of treatment modality. Further research is needed to explore clinical practices, the shared decision process and how socioeconomic factors influence the treatment of elderly patients with high-risk PCa.

## Introduction

Prostate cancer (PCa) is the second most commonly diagnosed cancer among men worldwide, affecting mainly older men [1–3]. In Europe, 50% of PCa patients are 70 years or older at time of diagnosis. Due to population aging, this proportion is estimated to increase to 60% by 2040 [4].

According to international guidelines, patients with high-risk PCa, and life expectancy > 10 years, should be treated with curative intent – either with radical prostatectomy (RP) or radiotherapy [5]. However, since elderly patients often are underrepresented in clinical trials [6], the International Society of Geriatric Oncology (SIOG) recommend that treatment of older patients (> 70 years) should be based on the patient’s health status and not on age alone [7]. Nevertheless, several studies have suggested undertreatment of older PCa patients [8–13]. With the lack of specific treatment recommendations for elderly PCa patients, health status assessment of this patient group may be a source of variation in clinical practice and subsequent disparity in cancer outcomes.

Geographic variation in curative treatment of PCa is well-documented. Numerous studies have shown variation in the choice of treatment due to regional differences [14–17], where several found an association between access to treatment and choice of treatment [18–22]. In Norway, the Norwegian Prostate Cancer Registry (NoPCR) has reported regional variation in the use of both RP and radiotherapy [23]. Other studies have found geographical variation in overall curative treatment [24–26]. The geographic variation in these studies was based on the place of residence of patients, treating hospitals or region density (urban versus rural). Socioeconomic variation in treatment of PCa has also been found where e.g. wealthy and/or highly educated men have higher odds of curative treatment and higher odds of receiving RP over radiotherapy [27–29].

There is however a paucity of information on geographic and socioeconomic variation in treatment of older PCa patients. The aim of this study was to examine whether there are geographic or socioeconomic variation in the treatment of elderly patients with high-risk PCa in Norway, both in relation to overall curative treatment and choice of treatment modality.

## Methods

### Study design and data sources

This cohort study included all Norwegian men diagnosed with non-metastatic, high-risk PCa between 2011 and 2020 at the age 70 years or older. Individual-level data were obtained from mandatory health and administrative registries with national and complete coverage. The data were linked by encrypted serial numbers derived from the personal identity number held by all Norwegian citizens.

The Cancer Registry of Norway (CRN) identified all older men with a PCa diagnosis (ICD-10 code C61) during the inclusion period and provided data on cancer diagnosis, diagnosis date, age, stage, and basis for diagnosis. Data on radiotherapy dates and doses were obtained from the national radiotherapy database. Information on functional status, diagnostic data, including Prostate-Specific Antigen (PSA), Gleason score, and tumor-node-metastatsis (TNM) status, and surgical data were received from NoPCR, the national quality registry on prostate cancer.

Data on health services provided at the primary care level by general practitioners and out-of-hours services were obtained from the Norwegian Control and Payment of Health Reimbursements Database (KUHR). Demographic and socioeconomic information came from Statistics Norway (SSB).

### Definitions

We defined high-risk PCa as PSA > 20 ng/mL, and/or Gleason score > 7, and/or clinical T-stage (cT) ≥ 2c, and/or clinical N-stage (cN) > 0 in accordance with the European Association of Urology (EAU) categorisation [5].

**Fig. 1:**
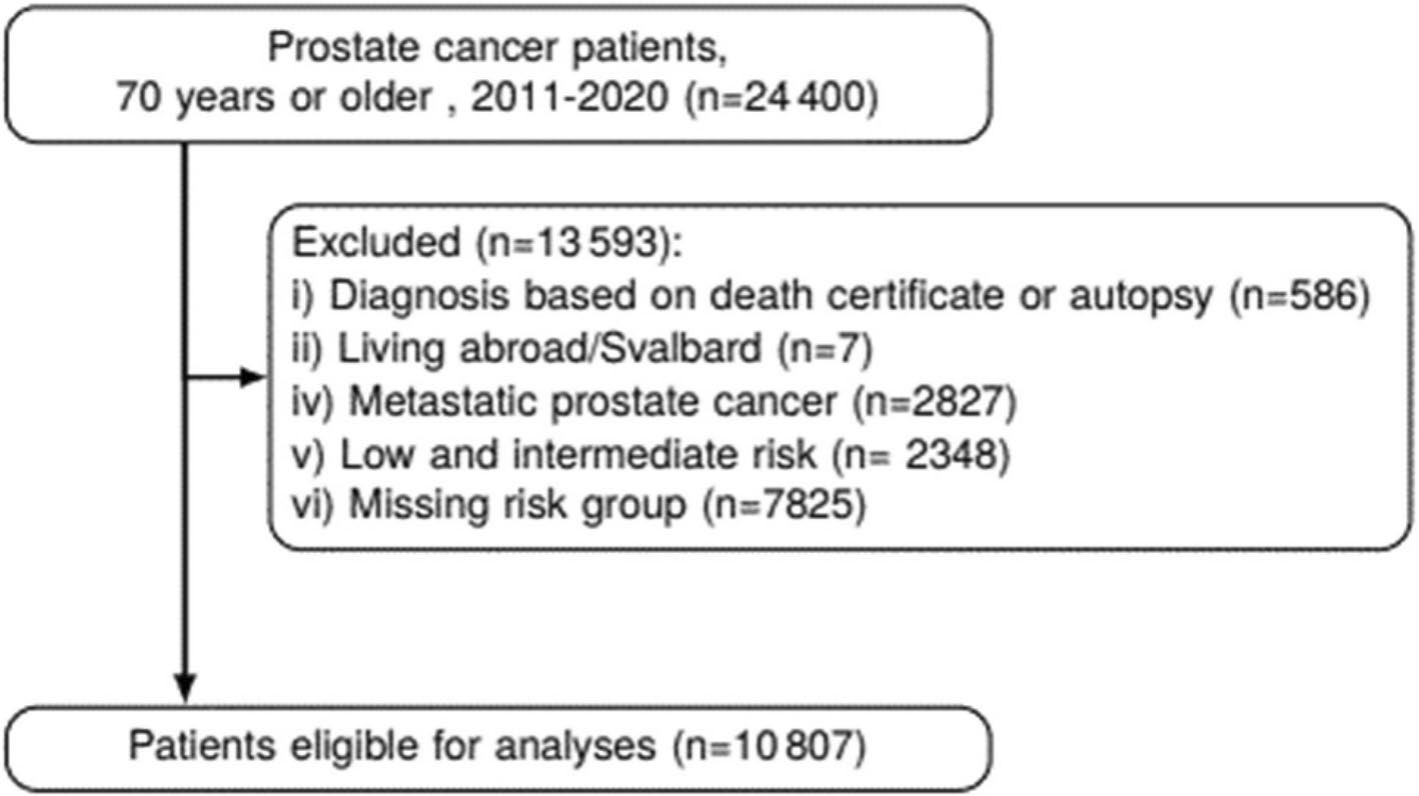
Flowchart showing inclusion of patients in the study

Patients with low or intermediate risk, metastatic PCa, or missing data for risk categorisation, were omitted from the study (Fig. 1). Additionally, individuals were excluded if their cancer diagnosis was based solely on death certificate or autopsy, or if they lived abroad.

The first binary outcome measure was receipt of curative treatment, either RP within six months or curative radiotherapy within 12 months of diagnosis. The second binary outcome was treatment modality, contrasting RP within six months to radiotherapy within 12 months. According to the Norwegian guidelines covering the inclusion period of the study, the recommended target dose of curative radiation was 66–78 Gy. We therefore defined curative radiotherapy as radiation with target dose ≥ 66 Gy. Treatment modality was classified as RP if patients received both RP and radiotherapy.

Public hospital trusts provide specialist health care services to the population living within their hospital referral area (HRA). In 2020, 13 hospitals performed RP, while eight radiation centres provided curative radiotherapy to PCa patients. Municipal residency in the year prior to PCa diagnosis defined the HRA affiliation of the patients.

Stage referred to extent of disease at the time of diagnosis, and was coded as localised, regional and unknown according to the CRN classification [30]. Frailty was measured by a frailty index (FI) based on primary care data [31]. We used the International Classification of Primary Care (ICPC) codes from KUHR within 12 months prior to the PCa diagnosis to calculate each individual FI score. FI was categorised into low (0–1), intermediate (2–3) and high score (≥ 4). Functional status was measured by Eastern Cooperative Oncology Group (ECOG) performance status and categorised into 0 (fully active), 1 (restricted in physically strenuous activity) and ≥ 2 (ambulatory and capable of all self-care, but unable to carry out any work activities, or worse).

Yearly after-tax personal income was calculated as the sum of income from employment, business, property and transfers received, including retirement pension from the National Insurance Scheme, minus assessed tax and negative transfers, in the year prior to the year of diagnosis. It was consumer price index adjusted and divided into quartiles (Q): low (Q1), intermediate low (Q2), intermediate high (Q3) and high (Q4) income.

A tripartition of the original nine educational levels into low, intermediate and high was according to the Norwegian Standard Classification of Education [32]. Low level was defined as less than high school (compulsory school grades 1–10), intermediate level corresponded to high school and high level to undergraduate and postgraduate education.

A household was regarded as all persons who lived permanently in the same dwelling and had common housekeeping. Type of household was categorised into living alone, not living alone and not living in private household. The latter category included persons who lived in nursing facilities.

Travel time by road to nearest hospital performing RP and nearest radiation centre was calculated in minutes from the municipal centre. Both variables were recoded into three categories; < 60, 60–120 and ≥ 120 min.

### Statistical analyses

Data were analysed using SAS V.9.4 (SAS Institute). Independent variables previously indicated by literature to be relevant for cancer treatment were considered for analysis. We used descriptive statistics for patient characteristics and conducted multilevel logistic regression analyses with hierarchical structured data (patients (individual level) clustered in HRAs (group level)) to estimate odds ratios (ORs) for comparisons across the HRAs and socioeconomic groups. Directed acyclic graphs (DAGs) was used to guide model selection for each analysis and reduce the risk of table 2 fallacy [33].

Empty models (null model) with cluster-specific random effects only were applied to model variation between HRAs. The intraclass correlation coefficient was calculated, detailing the proportion of the total variance in the outcome that was attributable to the HRA level [34]. The intraclass correlation coefficient at the HRA level was calculated by ${\sigma }^{2}/({\sigma }^{2}+\frac{{\pi }^{2}}{3})$ where ${\sigma }^{2}$ is the variance of the random intercept at the HRA level and ${\pi }^{2}$/3 is the assumed patient level error variance.

To assess whether any geographic variation was attributable to differences in patient characteristics, individual demographic and clinical factors (age, stage, FI, functional status, and also risk group in the analysis of treatment modality) were added to model 1, and additional socioeconomic factors (income, education, and type of household) to model 2. Travel time^1^ and year of diagnosis were included in model 3 to examine effects attributable to the HRA level. Additional factors such as patients’ children and their education and residential location were examined, but omitted due to non-significance and failure to improve the model.

All models were examined for multicollinearity by inspecting correlation and variance inflation factors.

## Results

In the period 2011–2020, 10 807 men aged 70 years or older were diagnosed with non-metastatic, high-risk PCa and eligible for inclusion in this study (Table 1).

**Table 1:**
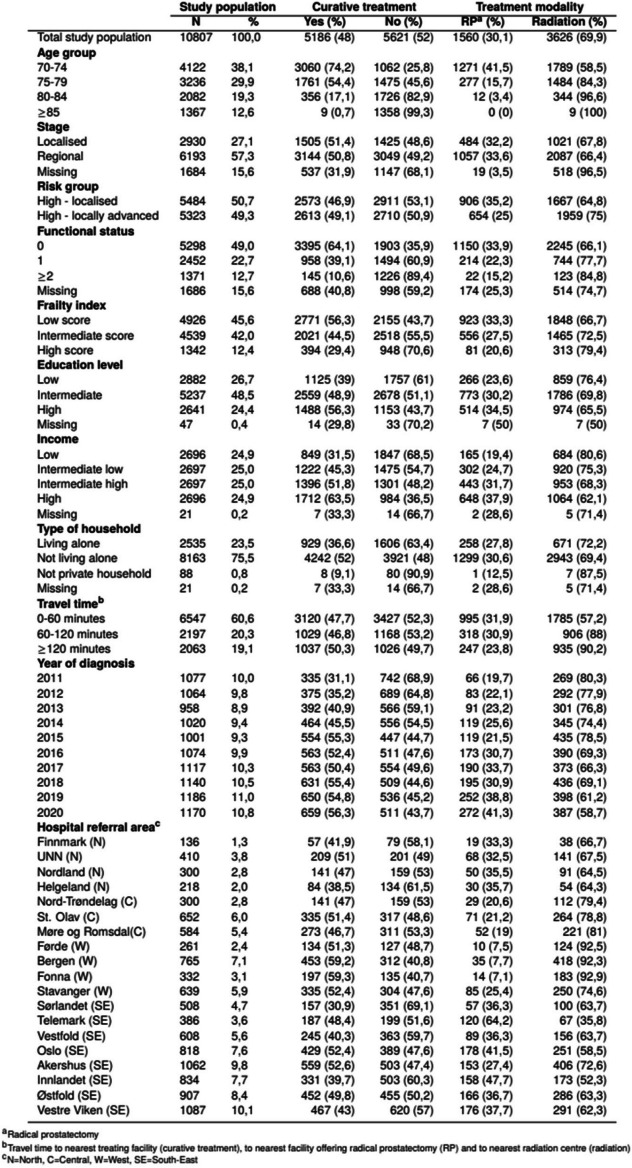
Characteristics of elderly patients diagnosed with high-risk prostate cancer in Norway in 2011–2020 according to type of treatment

**Fig. 2:**
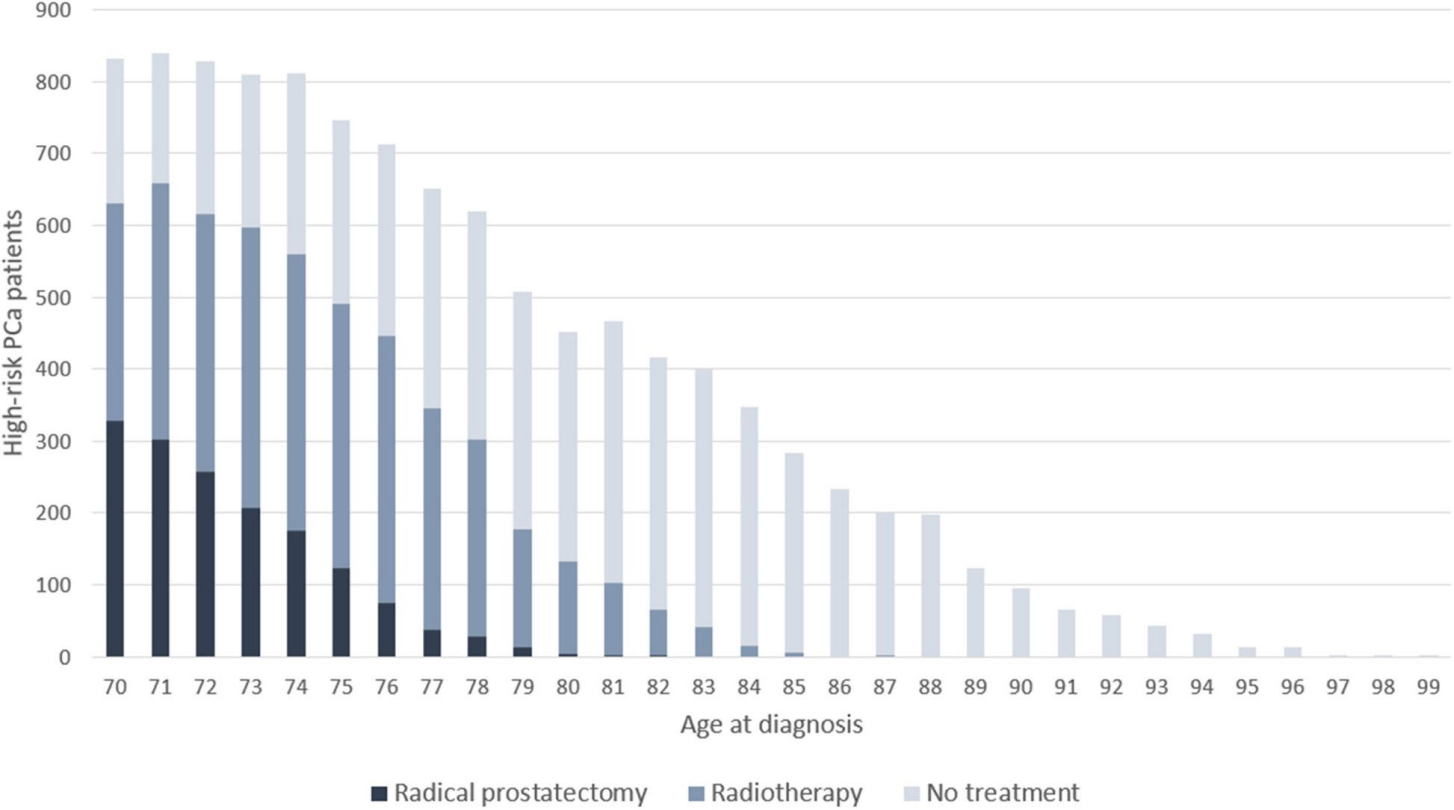
Number of patients diagnosed with high-risk prostate cancer by type of treatment at each age at diagnosis

Age ranged from 70 to 99 years at diagnosis (Fig. 2), with a mean age of 77.4. Less than half of the patients had low frailty score (46%). Half of the men (49%) had intermediate education level, while 27% had low education level. One out of four men lived alone. In total, 5186 patients (48%) were curatively treated with either RP or radiotherapy, ranging from 31 to 59% between the HRAs.

When comparing treatment modalities, 1560 (30%) of the treated patients received RP. Of these, 1271 (81%) were 70–74 years old, 277 (18%) were 75–79 years old and 12 (0.8%) were 80–84 years old. No patients ≥ 85 years received RP. The corresponding proportions of patients who received radiotherapy were 49%, 41% and 9%, respectively. Additionally, 9 patients aged ≥ 85 years were treated with radiotherapy. A larger proportion of RP-treated patients had high education and high income compared to those treated with radiotherapy (33% vs 27% and 37% vs 26% respectively).

### Geographic variation

**Fig. 3:**
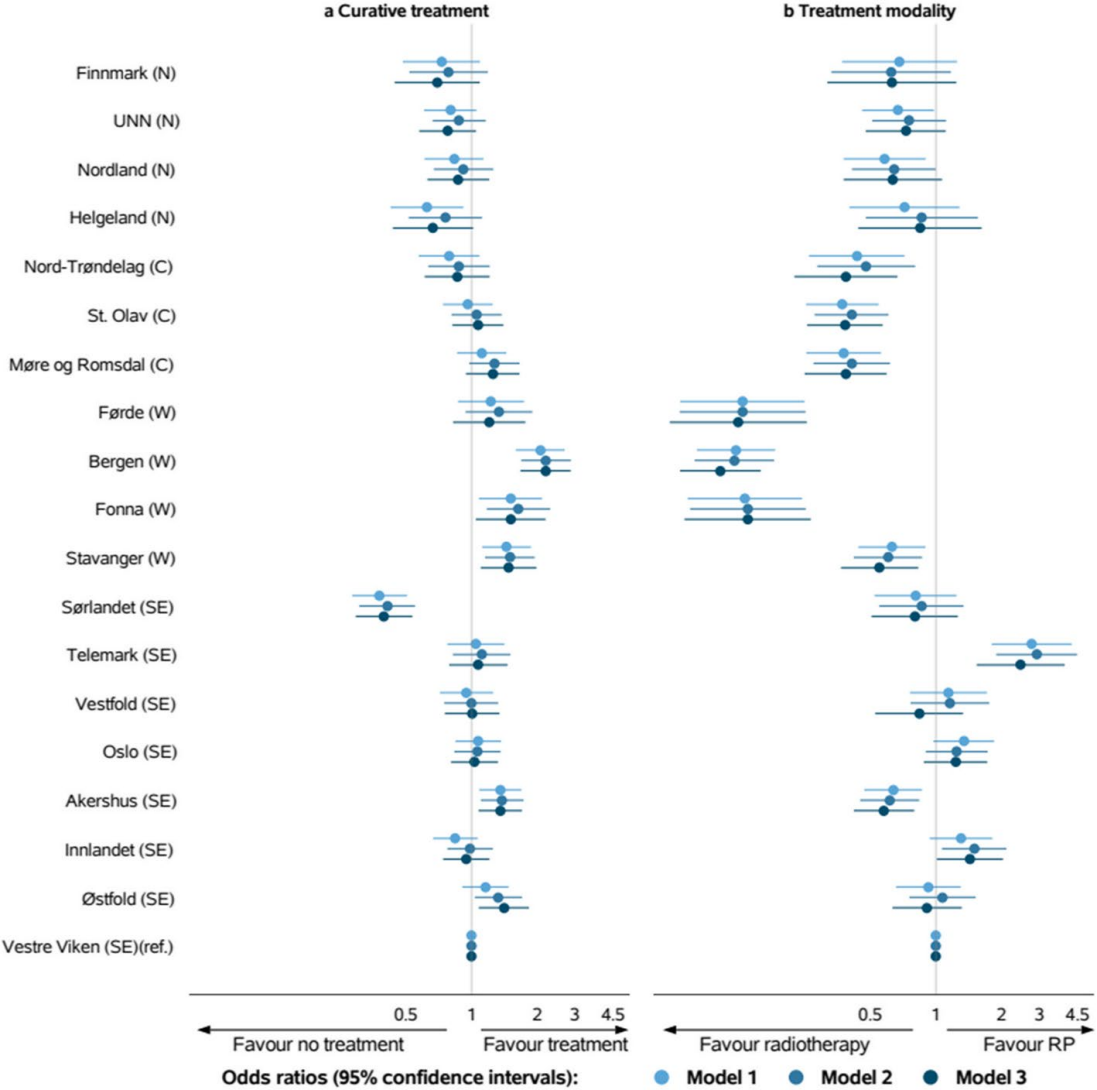
Odds ratios with 95% confidence intervals for a Curative treatment and b Treatment modality (radical prostatectomy vs radiotherapy) for each hospital referral areas grouped by the four regional health authorities: North (N), Central (C), West (W) and South-East (SE). Model 1: adjusted for demographic and clinical characteristics, Model 2: Model 1 + adjusted for socioeconomic factors, Model 3: Model 2 + adjusted for travel time and year of diagnosis

ORs with corresponding 95% confidence intervals (CIs)) for model 1, model 2 and model 3 with Vestre Viken as reference HRA are depicted in Fig. 3, separately for curative treatment over none, and for RP over radiotherapy. ORs with CIs for the HRAs for all models are described in Online Resource 1.

#### Curative treatment

Geographic variation in curative treatment across the HRAs were present in the null model. Moreover, the ORs remained largely unchanged when introducing clinical and demographic factors in model 1, socioeconomic factors in model 2, and accessibility in model 3. In the fully adjusted model, ORs ranged from 0.39 (95% CI 0.29–0.53) to 2.19 (95% CI 1.68–2.86) Patients in five HRAs had significantly higher odds for curative treatment than the reference HRA, whereas one HRA had lower odds. Only 4% of the total treatment variation was attributable to factors at the HRA level (Table 2).

Stratified analyses revealed a somewhat greater geographic variation in the older age groups. In the youngest group (70–74 years), 74% (HRA range 49–83%) of the patients received curative treatment. For the older groups (75–79 years and ≥ 80 years), a smaller proportion of patients received curative treatment: 54% (29–72%) and 11% (3–19%), respectively.

#### Treatment modality

We found substantial geographic variation in the use of RP versus radiotherapy (Fig. 3). The proportion of treated patients who had RP varied across HRAs from 7 to 64% (Table 1). Little change was seen in the ORs for treatment modality when expanding the regression model. ORs in the fully adjusted model ranged from 0.10 (95% CI 0.07–0.16) to 2.45 (95% CI 1.54–3.92) (Fig. 3). Patients in two HRA had significantly greater odds of receiving RP over radiotherapy than patients living in the reference HRA, whereas patients in eight HRAs had significantly lower odds. A substantial proportion of the total variation (18%) was attributable to factors at the HRA level.

Travel time to nearest radiation centre was associated with choice of treatment modality, but with conflicting results; Patients living 60–120 min from a radiation centre had higher odds of RP than radiotherapy compared to patients with travel time less than 60 min, while such association was not found for patients living more than 120 min from a radiation centre.

### Health and socioeconomic variation

At the individual level, increasing age, frailty and functional status were independently associated with decreasing odds of curative treatment (Table 2). In the fully adjusted model, patients aged 75–79 years had 56% lower odds of curative treatment than patients aged 70–74 years (OR 0.44 (95% CI 0.39–0.50)). The odds decreased further with increasing age. Odds for curative treatment decreased by 22% for patients with intermediate frailty score compared with patients with low score (OR 0.78 (95% CI 0.70–0.88)), and even more for patients with high frailty score. With a functional status of 1 the odds for curative treatment decreased by almost half compared to those with 0 (OR 0.56 (95% CI 0.49–0.64)). The odds decreased further for those with a functional status ≥ 2.

**Table 2:**
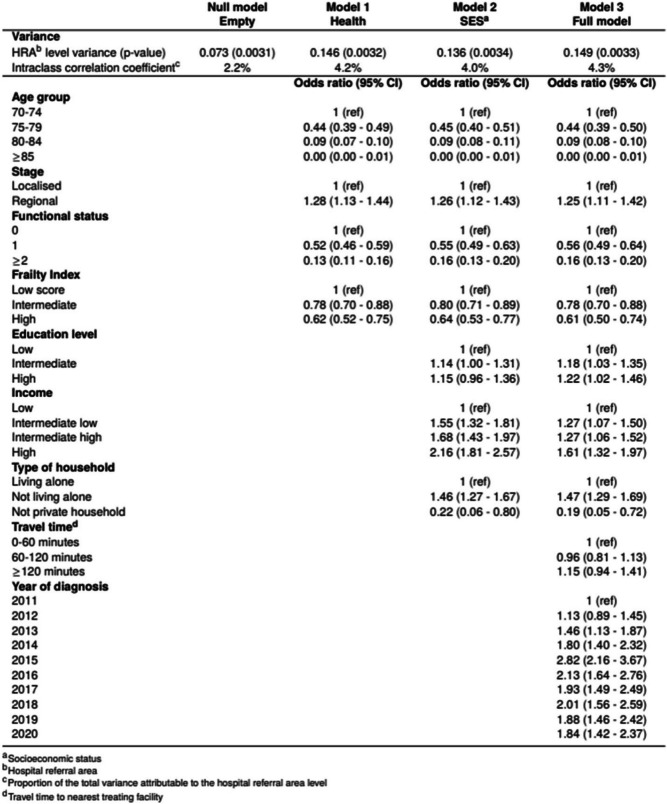
Multilevel logistic regression of four models with odds ratio and 95% confidence interval for curative treatment. Variance with *p*-value and intraclass correlation coefficient of hospital referral area. Model 1: adjusted for demographic and clinical characteristics, Model 2: Model 1 + adjusted for socioeconomic factors, Model 3: Model 2 + adjusted for travel time to nearest treating facility and year of diagnosis

Patients with higher income, higher education, and patients not living alone, were more frequently curative treated (Table 2). Patients with high income had 61% greater odds of being curative treated than those with low income (OR 1.61 (95% CI 1.32–1.97)), whereas patients with high education had 22% higher odds compared to patients with low (OR 1.22 (95% CI 1.02–1.46)). Men who did not live alone had 47% higher odds of receiving curative treatment compared to those living alone (OR 1.47 (95% CI 1.29–1.69)).

At the individual level, for patients who received curative treatment, older age was associated with lower odds of RP over radiotherapy. Patients aged 75–79 years had 76% lower odds of having RP compared to those aged 70–74 years (OR 0.24 (95% CI 0.20–0.28)) (Table 3). The odds decreased further with increasing age. Patients with high income had greater odds of having RP compared to those with low income (OR 1.72 (95% CI 1.28–2.32)).

**Table 3:**
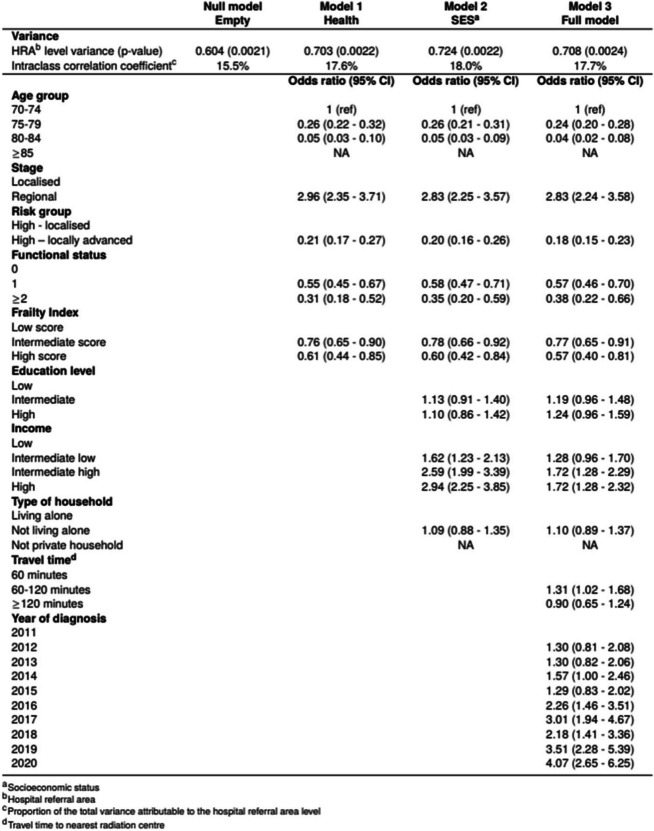
Multilevel logistic regression of four models with odds ratio and 95% confidence interval for choice of treatment modality (radical prostatectomy vs radiotherapy). Variance with *p*-value and intraclass correlation coefficient of hospital referral area. Model 1: adjusted for demographic and clinical characteristics, Model 2: Model 1 + adjusted for socioeconomic factors, Model 3: Model 2 + adjusted for travel time to nearest radiation centre and year of diagnosis

## Discussion

This study showed geographic variation in treatment of elderly patients with non-metastatic, high-risk PCa. Additionally, demographic and socioeconomic factors were associated with treatment, both curative treatment and choice of treatment modality. Few studies have explored variation in treatment of elderly patients with high-risk PCa. To our knowledge, this is the first study including individual data on both geographic and socioeconomic factors.

### Geographic variation

#### Curative treatment

Our study showed some geographical variation in curative treatment. This is in line with other studies showing geographic variation in Germany, the UK and the US [24, 25, 35]. At the same time, the intraclass correlation coefficient in our study was low, indicating that the geographic variation cannot be explained by factors on the HRA level, but rather would be due to individual features such as a physician’s or patient’s preferences.

#### Treatment modality

We found substantial geographic variation in choice of treatment modality, where many HRAs had lower odds of receiving RP over radiotherapy. Regional differences in choice of treatment have been documented in the Netherlands, the UK and the US [15, 16, 19, 21, 36]. Cooperberg et al. [36] showed substantial variation in treatment selection for localised PCa across 36 clinical sites in the US that was not explained by case-mix or known patient factors. The choice of treatment could impact quality of life. Common side-effects are disrupted urinary, bowel, and sexual functioning, where RP has been associated with higher risk of incontinence, but lower risk of bowel problems compared to radiotherapy [37, 38].

This study showed an association between travel time to nearest radiation centre and choice of treatment, but with conflicting results. Other studies have found associations between access to treatment and choice of PCa treatment [20, 22]. Muralidhar et al. [18] found that PCa patients were less likely to receive radiotherapy the farther away from a radiation centre they lived. When eligible for either RP or radiotherapy, patients need to consider time consumption of each modality when deciding on treatment. While RP is a short in-patient stay, radiotherapy occurs daily over several days and might be a less attractive option if it is too time demanding. Patients receiving radiotherapy might be staying overnight if the travel distance is considerable, which could explain the non-significance association between travel time ≥ 120 min and choice of treatment.

Our study showed that the use of radiotherapy was greater especially in patients within the Western region. Clinicians in Norway changed their treatment policies after studies regarding the effect of radiotherapy combined with hormonal treatment were published in the 1990s [39, 40]. This appeared especially in the Western region, where there was a more frequent use of radiotherapy since the mid-1990s [41]. It seems that the Western region has continued its practice since then.

Including patient characteristics and socioeconomic factors in the model did not explain the geographic variation in treatment modality. This variation might therefore be related to clinical practice. PCa management is regarded as preference-sensitive care where both RP and radiotherapy are considered valid curative treatments for patients with high-risk PCa [42–45]. Understanding the side-effects are therefore crucial when deciding on treatment. Shared decisions between patients and physicians are thus emphasised in PCa guidelines. However, Wennberg stated that “medical opinion rather than patient preference tends to dominate the treatment choice” in preference-sensitive care. This is in accordance with a systematic review indicating that physicians’ recommendations were the most consistent factor for why older adults accepted cancer treatment [46]. Moreover, Cohen and Britten [47] reported that treatment of men diagnosed with localised PCa was mostly decided by the clinicians.

### Age and frailty

This study showed that men of older age and higher frailty score had independently decreasing odds of receiving curative treatment compared to those of younger age and low frailty. These findings have also been documented in other studies [35, 36] and are in line with the guidelines which states that patients with a life expectancy < 10 years and/or high comorbidity are not directly eligible for curative treatment. A decrease in odds of curative treatment due to both age and frailty was therefore expected.

### Socioeconomic variation

Our study found that income and education was associated with curative treatment; Older men with lower income and lower education were independently less likely to receive curative treatment. Socioeconomic differences in management of PCa patients of all ages have been discussed extensively in several studies [14, 27–29, 35]. Deprivation, low income and low educational level have been reported as factors associated with less treatment of PCa patients.

Health literacy might be an underlying factor of the observed association between socioeconomic status (SES) and curative treatment. Health literacy is the “ability to find, understand, and use information and services to inform health-related decisions and actions for themselves and others” [48] and has been demonstrated to follow a social gradient where e.g. patients with financial deprivation were more likely to have limited health literacy [49, 50]. This will affect the ability to engage in shared decision processes, where patients with inadequate health literacy may be less capable of understanding information given and using it to decide on treatment together with the physician.

Another factor affecting the association between SES and treatment could be doctor–patient communication. Several studies have shown a social gradient in communication where physicians gave less information to patients with lower SES than to those with high SES [51, 52]. Furthermore, communication with lower social class patients had a less participatory consulting style, resulting in a less adapted shared decision-making process. This gradient in communication could be explained by the patient’s communicative style: Patients with lower SES are less active when communicating with their physician, ask fewer questions and are less opinionated compared to those with higher SES [53]. Physicians may therefore presume that these patients are less independent, responsible and less likely to comply with treatment regime [54], which could affect their decision on providing radical treatment to this group of patients.

Our study found that men living alone were less likely to receive curative treatment than men living with a cohabitant. Studies have shown that marriage was associated with curative treatment in men with PCa [24, 55]. Cary et al. [24] found that married men had 67% greater odds of treatment compared to those who were not married. As people age, cognition declines, and the ability to maintain functional independence is harder to uphold [56]. Physicians might therefore be hesitant to provide radical treatment to patients with cognitive impairment who live by themselves.

### Limitations and strengths

This study has several limitations. Information on disease progression was not available. Categorisation of risk groups was therefore based on diagnostics at the time of diagnosis. Hence, patients who had a progression of the tumour from a lower risk group to a high-risk group were not included in the study. Increased use of new diagnostic methods, such as MR, and varying adoption of these methods might have led to bias in risk group categorisation. Additionally, guidelines evolved during the study period and, in 2015, cancer patient pathway (CPP) was implemented for PCa patients in Norway. In particular, CCP could have had an impact on the results. Nonetheless, Nilssen et al. [57] showed that increasing age indicated lower odds of being included in CPP for PCa patients ≥ 70 years in 2015 and 2016. Another limitation is the lack of information on patients’ preferences on treatment options and on the shared decision process in the management of PCa patients. Unwarranted variation refers to disparities that cannot be explained by randomness, illness or patients’ preferences [58]. Since our study do not include patients’ preferences, we cannot definitively assert that the findings are entirely unwarranted.

The major strength of this study is the use of individual-level data from national registries which are of high quality and completeness. This have allowed us to include important factors, like frailty and SES, at the individual level in the analyses. This provides us with unique information and widely representative results.

## Conclusion

Although Norway has a universal health care system set to provide equal health care regardless of place of residence and SES, this study demonstrated variation in treatment of older patients with high-risk PCa in Norway, both with regards to place of residence and SES, and that treatment management is not in line with Norwegian health policy. Further research is needed to explore clinical practices, the shared decision process and how socioeconomic factors influence the treatment of elderly patients with high-risk PCa.

## Data Availability

The data underlying this article cannot be shared publicly due to legal restrictions.

## Footnotes

1 Travel time to nearest treating facility (RP or radiotherapy) was included in the model with curative treatment as outcome, while travel time to nearest radiation centre was included where treatment modality was the outcome.
